# Acute Phase Proteins in Marine Mammals: State of Art, Perspectives and Challenges

**DOI:** 10.3389/fimmu.2019.01220

**Published:** 2019-05-29

**Authors:** Maria Elena Gelain, Federico Bonsembiante

**Affiliations:** ^1^Department of Comparative Biomedicine and Food Science (BCA), University of Padova, Padova, Italy; ^2^Department of Animal Medicine, Productions and Health, University of Padova, Padova, Italy

**Keywords:** marine mammals, immune system, acute phase reaction, acute phase proteins, serum proteins

## Abstract

The term “acute phase response” (APR) is referred to a nonspecific and complex reaction of an organism that occurs shortly after any tissue damage, such as infection, trauma, neoplasia, inflammation, and stress. The APR can be identified and monitored with some laboratory tests, such as the concentration of several plasma proteins, the acute phase proteins (APPs). The APPs are components of the non-specific innate immune response, and their plasma concentration is proportional to the severity and/or the extent of tissue damage. The evaluation of health status of marine mammals is difficult because the classical clinical signs of illness used for human and domestic animals are difficult to recognize and understand. For this reason, in the past years, several efforts were done to identify laboratory markers of disease in these animals. The APPs have demonstrated their role as early markers of inflammation in veterinary medicine, thus several APPs were tested in marine mammals, such as C-reactive protein (CRP), serum amyloid-A (SAA), and Haptoglobin (Hp). However, the difficulty to extrapolate the knowledge about APPs in one species to another, the lack of specie-specific reagents, the absence of data about negative APPs have hampered their extent use in marine mammals. Herein, the state of art of APPs in marine mammals is reviewed, with particular attention to pre-analytical and analytical factors that should be taken into account in validation and interpretation of APPs assays. Moreover, the current application, potential utility and the future developments of APPs in marine mammals is highlighted and discussed.

## Introduction

The mammalian immune system includes innate or nonspecific immunity as well as adaptive or specific immunity. The responses of these two different pathways are distinct, but highly interconnected. The first reaction of an organism to different pathological conditions is an innate, non-specific response (1), a more conserved response during evolution which aim is the immediate reaction against pathological stimuli (2). After the initial recognition of pathogens or tissue damages by the tissue-resident macrophages, which express the pattern recognition receptors (PRRs), a variety of different inflammatory mediators are produced by leukocytes, endothelial cells, tissue cells or are derived from plasma proteins. These mediators include different chemokine, cytokine, vasoactive amines and products of the arachidonic acid: their primary effect is to elicit inflammatory response locally and to recruit leukocytes and plasma proteins in the site of injury (3).

At the same time, many of these inflammatory mediators influence the function of many tissues and organs throughout all the organisms, inducing the acute phase response.

The activated macrophages on the site of injury are also responsible of the activation of the adaptive immune response, a more slowly, but more focused response. The expression of major histocompatibility complex (MHC) class II antigens, combined with foreign antigen, by tissue macrophages leads to the antigen-dependent activation of T-cells and start the precise immune response against that specific target. T-cells can differentiate in different kinds of T-helper (Th) lymphocytes with the expression of different cytokine pattern. Th1 cells coordinate the interaction between innate and cell-mediated adaptive response by means of different cytokines, among them IFN-γ and IL-2. Differently, Th2 cells, which produce IL-4 and IL-5, promote humoral immune response, the differentiation of B-cell into plasma cells, but have also an anti-inflammatory activity by producing IL-10 (4).

The term “acute phase response” (APR) refers to a nonspecific and complex reaction that occurs shortly after tissue damage due to several conditions such as infection, trauma, neoplasia, inflammation, and stress (5). The reaction of APR includes systemic consequences, such as fever, leucocytosis, increased release of several hormones and drastic rearrangement of plasma protein synthesis (6). These changes include a decrease of plasma low and high density lipoproteins-bound cholesterol, an increase of ACTH and glucocorticoids, the activation of complement and coagulation system, a decrease of calcium, zinc, iron, vitamin A, and α-tocopherol serum levels. All these changes in blood plasma composition induced by APR are beneficial to the organism because they prevent microbial growth and help restore of homeostasis (1). Moreover, a considerable change in concentration of several plasma proteins, the acute phase proteins (APPs), is evident few hours after an inflammatory stimulus (7).

APPs are a group of blood proteins that change in concentration in animals subjected to external or internal challenges, such as infection, inflammation, trauma or stress, proportionally to the severity of the disorder and/or the extent of tissue damage (8). They are synthesized primarily by hepatocytes stimulated by cytokines or endogenous glucocorticoids (9, 10). APPs are classified based on the direction of change: synthesis of positive APPs is upregulated with a resulting increase in blood concentration, and synthesis of negative APPs is downregulated with a resulting decrease in blood concentration (11). Positive APPs are further classified as major, moderate or minor based on the magnitude of increase: major APPs showed an increase of 10- to 100-fold, moderate APPs showed an increase of 2- to 10-fold, and minor APPs have only a slight increase (5). Also the rate of the concentration increase varies between major, moderate, and minor positive APPs: major APPs usually have a marked increase within 48 h after a pathogen stimulus and a subsequent rapid decline after the cessation of stimulus due to their short half-life (12). Moderate and minor APPs usually have a slower increase, but also a slower decline to normal value and so they usually increase during chronic inflammatory process (5). However, the APPs response varies between species: the major APP in dogs and men is C-reactive protein (CRP) (13), in cats is α1–acid glycoprotein (AGP) (9), in ruminants is Haptoglobin (Hp) (14); in horse is Serum Amyloid A (SAA) (15) and in pigs are Hp, SAA, and major acute phase protein (MAP) (10).

Recently, in veterinary medicine, studies on the role of APPs as markers of infectious, inflammatory and neoplastic diseases have proliferated (16) and at least 40 different plasma proteins have been identified as APPs (8). Their use as marker of homeostasis perturbation provides some advantages compared with traditional parameters like the white blood cell (WBC) counts. Compared to WBC count, the diagnostic sensitivity of APPs is higher and the change in concentration is faster (17). Moreover, their stability in serum/plasma is high, so it is possible to measure APPs in frozen samples (18). One limitation of the APPs is the poor diagnostic specificity, for this reason they cannot be used as primary diagnostic test for a specific disease, but they were successfully used to detect subclinical diseases and to monitor clinical evolution and to assess the response to treatment (5). Additionally, the combined measurement of several APPs provides more information than the evaluation of a single protein: in the “APP value index,” proposed by Gruys et al. (1), sensitivity and specificity are improved by combining response of both positive and negative major and minor APPs.

Marine mammals are a group of around 130 mammalian species which depend on water environment for most of their needs. They include 3 orders: Carnivora (pinnipeds, otters, and polar bear), Cetacea (dolphins, whale, and porpoise) and Sirenia (manatee and dugogons). Marine mammals are differently adapted to life in water, with some species, which are fully aquatic (cetaceans and sirenia), and others that spend part of their life on land (pinniped and polar bears) (19). From an immunological point of view, aquatic adaptation caused few differences in distribution and function of immune system between marine and terrestrial mammals (20). However, nowadays the marine mammal' immune system is deeply exposed to environmental pollution because they are a long-lived animals placed at the top of food chain, thus they are exposed to a progressive bioaccumulation of fat-soluble pollutants, such as PCBs, which affect both innate and adaptive immune function (21). For these reasons, increasing knowledge in cellular and humoral immune response is continuously required to understand their immune system and in particular its relationship with infectious pathologies and the environmental pollution (22). Furthermore, marine mammals live also in controlled environment like aquaria, rehabilitation facilities and research center where health assessment is fundamental to evaluate the correct management of animals and to monitor the response to therapy during rehabilitation. From this perspective, the availability of new markers to asses immune functions is fundamental both for medical care and research purpose (20).

## Acute Phase Reaction and Acute Phase Protein in Marine Mammals

Innate immune response represent the first line of response against pathological stimuli, it's very fast and it's based primarily on effector cells (e.g., mast cells, macrophages, neutrophils) and antimicrobial substances (e.g., complement, reactive oxygen, and nitrogen species). On the other hand, adaptive immune system is antigen-specific, it takes more days to be effective and it's based on different T-cells response and on B-lymphocytes which are responsible of humoral response mediated by the different subclass of immunoglobulin (IgG, IgM, IgA) (20). Several assays were proposed to evaluate both immune response in marine mammals, generally based on isolated leucocytes with the aim to evaluate the leukocytes response against *in vitro* stimuli (23).

To assess the response pattern of cetaceans' cellular innate immune system, the phagocytosis and the generation of reactive oxygen species of polymorphonuclear leukocytes were investigated. In particular, *in vitro* ingestion of latex beads and hydrogen peroxide production have been evaluated in beluga whales (*Delphinapterus leucas*) and in bottlenose dolphins (*Tursiops truncatus*) (24, 25) whereas phagocytosis and respiratory burst assay, using whole blood from bottlenose dolphins, were used to assess the antimicrobial activity (26). In addition, the investigation of APR by analyzing the cytokine expression gives important information on the functionality of lymphoid cells. The production of specie-specific antibodies allows the development of immunological assays for the quantification of cytokine expression useful to investigate the inflammatory response in whales and dolphins. The coding regions of IL-2, IL-1β, IL-6, and TNF-α gene of the beluga whale have been sequenced, and a cytokine-specific rabbit antisera have been produced (27–29). In harbor porpoise (*Phocoena phocoena*), the quantification of mRNA of IL-1β, IL-2, IL-4, IL-6, IL-10, and TNF-α have been performed by RT-PCR (30), and the increase of IL-10 was seen in harbor porpoises suffering from long lasting infectious (31). Also in bottlenose dolphins, pacific white-sided dolphins (*Lagenorhynchus obliquidens*), and beluga whales, IL-2, IL-4, IL-10, IL-12, IL-13, IL-18, TNF-α, TGF-β, and interferon (IFN)-γ quantification was performed using RT-PCR (32). An IL-2 receptor expression assay and an IL-6 ELISA were developed in bottlenose dolphins and killer whale (*Orcinus Orca*), respectively (33, 34).

Similarly, both innate and the cell-mediated response were studied in pinnipeds. To better understand the innate response, phagocytic activity of isolated peripheral blood leukocytes was evaluated in harbor seal (*Phoca vitulina*), gray seal (*Halichoerus grypus*), and harp seal (*Phoca groenlandica*) pups, in harbor seal female during lactation and in harbor seal pups admitted to rescue center (35, 36). The authors found an age-related variation in both pups and adults: phagocytosis increased with age in gray and harbor seal pups, while in female harbor seals decreased from sub-adult to adulthood. At the same time, pups after rehabilitation showed a decreased phagocytic activity, probably due to the decreased stimulation of innate response after therapy. Also cytokine response was evaluate in harbor seal. Pro-inflammatory cytokine mRNA (IL-1β, IL-6, IL-8, and IL-12) in pups in a rehabilitation center were higher at admission whilst IL-4 was higher before the release (37), demonstrating the recovery from inflammation. Recently, a multiplex canine cytokine assay was validate in harbor, gray and harp seal to measure proteins levels in cell culture supernatant of peripheral blood mononuclear cells (PBMC) (38).

However, all these techniques are not generally applicable in a clinical setting in which the primary goal is a sensitive diagnostic tool with a rapid turnaround, even if give us important information on factors affecting cetaceans' immune system. For this reason, in the past years, several efforts were made to identify laboratory markers of disease in these animals. First parameters tested were WBC and erythrocyte sedimentation rate (39). However, even if they are inexpensive and rapid, they lack specificity and sensitivity. Moreover, changes in WBC occur after several hours after inflammatory stimuli. Thus, efforts were directed to identify inflammation at earlier stage (40). To examine the humoral response, species-specific antibodies against IgG were produced and used to evaluate serum IgG levels in killer whale by radial immunodiffusion assay (41) and by competitive ELISA in bottlenose dolphins (42, 43). The determination of IgG baseline values in free-ranging and in managed dolphins revealed higher levels of immunoglobulin in the first group with several values over the accurate range of the assay, probably due to the higher parasitic load in free-ranging dolphins (43).

Serum total protein, albumin, globulin and albumin:globulin ratio (A:G) are undoubtedly among the most measured markers in basic health assessment in domestic animals as well as in marine mammals. Serum protein electrophoresis is also broadly applied in veterinary medicine and it has the advantage to produce an accurate measurement of albumin and the visualization of globulin fractions (44). The interpretation of total proteins values and electrophoretic pattern of serum proteins is receiving increased attention also in marine mammals in which a typical pathologic pattern could be identified in inflammatory diseases (40). Reference intervals for these markers are available for free-ranging bottlenose dolphins (45) and, compared to these, recently data on managed dolphins showed slightly lower values of TP, α-globulins, and γ-globulins and higher albumin and albumin/globulins ratio (46).

It's interesting to note that Hp, α1-antitrypsin, α1-antichymotripsin, and α2-macroglobulin migrate in the α-globulins fraction, while the IgG and CRP migrate in the γ-globulins fraction. Albumin acts as a negative acute phase protein since the synthesis of this protein is decreased during an inflammation (47). Thus, the lower concentration of positive APPs associated to a higher concentration of albumin and the consequent higher albumin/globulins ratio could reflect lower antigenic stimuli in managed population compared to the free-ranging populations (36). Serum total protein analysis were used to assess health status in several cetaceans species such as pantropical spotted dolphins (*Stenella attenuata*) (48), beluga (49), minke whales (*Balaenoptera acutorostrata*) (50) and killer whales (51) as well as in other marine mammals, like harbor seals (*Phoca vitulina*) (52) and walruses (*Odobenus rosmarus*) (53). In all these species, serum total protein analysis was demonstrated to be one of the most used and commonly accepted marker of inflammation.

However, specific APPs have demonstrated their superior role as early markers of inflammation, so based on the results obtained in humans and companion animals, several positive APPs were tested in marine mammals (Table 1). Published works had the primary aims to evaluate the feasibility of the assays to measure the APPs, to validate the antibody-based assay and to determine the RIs. In bottlenose dolphins three APPs (CRP, SAA, and Hp) were tested, even if not always complete validation studies were performed (54, 61). For these APPs, the authors established the RIs in free-ranging and managed dolphins using automated assays (54) and they found significantly lower SAA and higher Hp levels in free ranging animals. The only clinical significance of these alteration was a higher ability to detect chronic inflammation for Hp. Regarding Hp, Segawa and colleagues validated commercially available Hp-ELISA and Hp-hemoglobin binding assay in bottlenose dolphins with ‘’acceptable” intra- and inter-assay imprecision (CV: 3.3% healthy dolphins and 3.5% inflamed dolphins; CV: 10.4% healthy dolphins and 21.7% inflamed dolphins) and demonstrated that Hp levels in the serum increase under inflammatory conditions (62).

**Table 1:** Acute phase proteins: site of production, role and marine mammal species in which they are validated.

**Acute phase protein**	**Site of production**	**Role**	**Positive/negative**	**MAJOR APP IN**	**Validated in**	**References**
CRP	Hepatocytes	Complement activation and opsonisation; modulation of monocytes and macrophages; cytokine production; binding of chromatin; prevention of tissue migration of neutrophils	Positive	DOG, HUMAN	Bottlenose dolphin; Harbor porpoise; Harbor seal	Cray et al. (54); Fonfara et al. (37); Müller et al. (55)
SAA	Hepatocytes	Transport of cholesterol from dying cells to hepatocytes; inhibitory effect on fever; inhibitory effect on the oxidative burst of neutrophilic granulocytes; inhibitory effect on *in vitro* immune response; chemotaxic effect on monocytes; polymorphonuclear leucocytes and T lymphocytes; induction of calcium mobilization by monocytes; inhibition of platelet activation	Positive	HORSE, PIG	Bottlenose dolphin; West Indian manatee	Cray et al. (54); Harr et al. (56); Cray et al. (57)
Hp	Hepatocytes	Binds hemoglobin dimers so that iron is not available to organisms; bacteriostatic effect; stimulation of angiogenesis; role in lipid metabolism; immunomodulatory effect; inhibition of neutrophil respiratory burst activity	Positive	COW, PIG	Bottlenose dolphin; Harbor porpoise; Harbor seal; Stellar sea lion; Ringed seal; West Indian manatee	Cray et al. (54); Frouin et al. (35); Fonfara et al. (37); Harr et al. (56); Müller et al. (55); Zenteno-Savin et al. (58); Rosenfeld et al. (59)
AGP	Hepatocytes	Several anti-inflammatory activities	Positive	CAT		Not validated
fibrinogen	Hepatocytes	Hemostasis	Positive		Bottlenose dolphin; West Indian manatee	Terasawa et al. (60); Harr et al. (56)
albumin	Hepatocytes	Major contributor to oncotic pressure, transports Ca2+, Mg2+, unconjugated bilirubin, fatty acids, thyroxine, and many other substances	Negative		Bottlenose dolphin; Spotted dolphin; White whales; Minke whales; Killer whales; Harbor Seals; North Atlantic walrus	Schwacke et al. (45); Gili et al. (46); St. Aubun et al. (48); Tryland et al. (49); Tryland and Brun (50); Robeck and Nollens (51); Greig et al. (52); Tryland et al. (53)
PON	Hepatocytes	Protection against oxidative stress; protection against organophosphate compounds	Negative			Not validated

Positive APPs were tested also in Florida manatees (*Trichechus manatus latirostris*) to define the more accurate marker of inflammation. Five different APPs were tested: AGP, CRP, Hp, fibrinogen, and SAA. SAA showed the highest diagnostic sensitivity and specificity (90% for both sensitivity and specificity) in the detection of inflammatory diseases, the diagnostic specificity of Hp and fibrinogen were 93 and 95%, respectively, while their diagnostic sensitivity were 60 and 40%, respectively, (56). When used in stranded manatee suffering from cold stress and trauma, SAA showed 93% of sensitivity and 98% of specificity in detecting diseased animals (57). By contrast, the Abs used for the determination of AGP and CRP did not cross-react in this species (56).

In harbor seal, an Ab anti-CRP and a competitive immunoassay was produced (63), but Hp is probably the APP most used in pinnipeds. A multispecies assay based on hemoglobin binding capacity was used to demonstrate as Hp is a sensitive marker of the health vs. disease status in harbor seal (64). In seal pups admitted in a rescue center. Hp, total protein, IgG and globulin values correlated positively, but Hp levels increased during the hospitalization, probably reflecting age-related changes (35). Hp is considered a health marker also in Steller sea lions (*Eumetopias jubatus*): significantly higher levels of Hp were found in declining population compared to more stable ones (58). However, also genetic differences between distant and isolated population of wild animals could be the causes of this difference, not only a pathological condition.

If some data on marine mammals positive APPs are available in literature, quite surprising are the lack of data available on negative APPs. For these reasons, the possibility to evaluate the usefulness of an “APP value index” is still far from being applied.

## Acute Phase Protein in Marine Mammals: Challenges and Future Developments

The availability of sensitive markers of inflammation both for free-ranging and managed marine mammals is nowadays considered fundamental to evaluate the health status and, in rehabilitation setting, to monitor the response to therapy and to define the prognosis. As serum markers, the APPs have several advantages: they have longer stability compared to other blood component such as WBC; they can be performed on frozen serum, thus the samples can be shipped to references laboratories; some assays can be automated to obtain results in an excellent turnaround time.

However, is important to consider that the knowledge about APPs in one species cannot be readily generalized to another species, in which healthy levels, response to inflammation or infection, and prognostic significance may be different (65). Moreover, the evolution of marine mammals and their adaptation throughout the millennia to an aquatic environment had led to a different physiology and metabolism compared to terrestrial mammals. Thus, the understanding of the genetic, phenotypical and biochemical properties of marine mammals APPs are essential prior to using them as a new biomarker.

An example of how the biochemical properties influence the analytic method is paroxonase-1 (PON1), a HDL-bound esterase which protects against organophosphate compounds, acts as negative APP and as oxidative stress marker. PON1 is usually assessed by enzymatic method and, based on the different PON1 functions, several substrates have been identified to evaluate serum PON1 activities. Nevertheless, both in humans as in some terrestrial mammals, PON1 gene polymorphisms highly influence the enzymatic activity toward different substrate: the single-nucleotide polymorphisms (SNPs) Leu55Met and Gln192Arg increase the paraoxonase activity (66) in humans and different PON1 genotypes influence activities toward paraoxon and phenyl-acetate in rabbit (67). Also in cows, some SNPs in the promotor region of PON1 gene are associated with serum PON1 activity (68). Recently, a phylogenetic study on convergent functional losses across marine mammals, has identified a PON1 functional loss in marine mammals, probably related to their different lipid metabolism and fatty acid oxidation due to adaptation to the marine environment and a high concentration of ω-3 fatty acids on their diet. As a consequence, in several marine mammals species paroxonase activity is very low, while enzymatic activity against other PON1 substrates is still present, such as arylesterase activity (69). For these reasons, the use of classical enzymatic assays is hampered in these animals and further studies are needed to elucidate the role of PON1 as possible negative APP, oxidative stress marker and the consequences of its inability to detoxify organophosphates compounds.

From an analytical point of view, another challenge in the evaluation of APPs in marine mammals is the need of species-specific assays, especially for the immunological assay, such as ELISA or immunoturbidimetry. This means the development of a *de-novo* method, often a time-consuming and expensive approach, or the validation of a commercial available assay used in other species (65). The latter approach is surely the most used in veterinary medicine, in which some human assays were validated for dogs, cats and horses (62). However, even if some APPs appeared highly conserved among species, an accurate validation of antibody cross reactivity is needed as well as species specific standards and control material (54). Among positive APPs, SAA is the most used across different species: it appeared as the most conserved APP in mammals even if some difference in circulating isoforms were reported (61) and it's considered a major APP in all the mammals in which it was investigated (65). Some commercial SAA assays showed good results also in marine mammals, such as bottlenose dolphin, manatee and striped dolphin *(Stenella ceoreloualba)* (54, 56, 70) and its use as diagnostic and prognostic marker appears nowadays the most promising.

To obtain accurate data, all the pre-analytical factors that could influence the results should be taken into consideration. The effect of storage, temperature and different anticoagulant had to be evaluated in a correct validation process as well as the interference of hemolysis and lipemia, as done in other species (5).

The application of a novel biomarker required a full evaluation of all the analytical performances and the clinical value. This process is usually divided in 4 steps: the assessment of analytical features (precision, accuracy, detection limits), the overlap performance (the ability to detect difference between healthy and diseased animals), the assessment of diagnostic capacity (sensitivity, specificity, accuracy, positive, and negative predictive values) and, at the end, the evaluation of the outcome of the new methods (which is the advantage of the test and its influence in the patient management) (65). In veterinary medicine, the validation studies do not always follow all these steps, mainly due to the lack of resources or technical limitation (44, 65). Also in marine mammals, the majority of studies had performed only some steps (44, 54, 56, 61, 62). This is mainly due to the limitation in species-specific reagents, the number of samples from animal with known health status and, last but not least, the capability to generate appropriate reference intervals, hampered the possibility to perform complete validation studies.

Population-based reference intervals derived from an appropriate group of reference individuals are usually required for diagnostic purpose (71). However, a number of biological factors have to be taken in consideration to select the appropriate reference population. Surely, age, sex and pregnancy could be used for partitioning (45, 72), but in marine mammals greater attention should be given to the difference between wild and managed animals. Serum protein electrophoresis values obtained in managed bottlenose dolphins showed lower total proteins and higher albumin levels compared to reference intervals derived from free-ranging (46) while 9 wild-caught manatees, apparently clinically healthy, had SAA level above reference limit (56). These data could indicate a trend to an inflammatory status or the presence of subclinical inflammation in free-ranging animals which are more exposed to immunological stimuli. In any case, this highlights the need to define appropriate reference intervals for animals living in different environment to have an accurate toll for the evaluation of clinical condition.

Compared to human and companion animals, the use of APPs in marine mammals is just getting started. The increasing need of knowledge on immune system and its response against infectious diseases or chemical pollutants and the request of more sensitive inflammation markers have increased the effort of researchers to study the APR and APPs. Even if APPs are considered a sensitive, but non-specific marker of inflammation, some studies revealed that, in some infectious diseases, APPs showed a specific behavior and biochemical features. One example is the modification of the glycan moiety of AGP in feline infectious peritonitis, FIV and FeLV, influencing the host-pathogens interaction and the immune response (73–75). Currently, some of the greatest threats for wild marine mammals is pathogens, like *Morbillivirus, Herpesvirus, Brucella ceti*, and *Toxoplasma gondii* (76): the evaluation of APR and APPs patterns during these infectious diseases could lead to the identification of a distinctive response of the immune system and increase the understanding of host-pathogen interaction.

Secondly, for managed or rescued animals, the forthcoming needs are the increase of automated assays, the standardization of procedures across laboratories and the discovery of new markers, for example negative APPs, to generate an APP index also in marine mammals. These new tools will certainly increase the diagnostic and prognostic skills for health assessment and, especially for stranded animals, the development of new “health status” markers will provide valuable resources in evaluating the response to treatment and rehabilitation prior to the release into the wild.

## Author Contributions

MG and FB analyzed the literature review, designed, and wrote the review.

### Conflict of Interest Statement

The authors declare that the research was conducted in the absence of any commercial or financial relationships that could be construed as a potential conflict of interest.
